# Molecular detection of Metallo-Beta-Lactamase and alginate in multidrug resistance *Pseudomonas aeruginosa* isolated from the clinical specimen

**DOI:** 10.25122/jml-2021-0196

**Published:** 2022-09

**Authors:** Govend Musa Qader, Khanzad Khudhur Jarjees, Rozhhalat Khudhur Jarjees

**Affiliations:** 1 Department of Biology, College of Science, University of Salahaddin-Erbil, Kurdistan Region, Iraq; 2 Department of Food Technology, College of Agricultural Engineering Sciences, University of Salahaddin-Erbil, Kurdistan Region, Iraq; 3 Department of Pharmacy, Erbil Medical Technical Institute, Erbil Polytechnic University, Erbil, Kurdistan Region, Iraq

**Keywords:** Alginate, *Pseudomonas aeruginosa*, Hemolysin, Metallo-beta-lactamase, Imipenem resistance isolates, AMK – Amikacin, CAZ – Ceftazidime, CIP – Ciprofloxacin, CLSI – Clinical and Laboratory Standards Institute, ESBL – Extended Spectrum Beta-Lactamase, GEN – Gentamicin, IPM – Imipenem, LPS – Lipopolysaccharide, MBLs – Metallo-β-lactamases, MDR – Multidrug Resistant, PCR – Polymerase Chain Reaction, PIP – Piperacillin, RND – Resistance-Nodulation-Division, TSB – Tryptic Soy Broth, TTSS – Type III Secretion System

## Abstract

*Pseudomonas aeruginosa* pathogen is opportunistic. Several virulence factors and biofilms can cause its pathogenicity. Furthermore, infections triggered via multidrug-resistant *P. aeruginosa* among hospitalized patients are a public health concern. The primary antimicrobial agents in treating Gram-negative infection include Meropenem and Imipenem. Moreover, the spread of Carbapenem-resistant *P. aeruginosa* is a focal concern worldwide. The present research aims to determine the spread of Carbapenem-resistant *P. aeruginosa*, and the distribution of the Alginate and Metallo-beta-lactamase encoding gene in clinical isolates. In the present cross-sectional descriptive research, 50 wound and sputum clinical specimens were obtained. Isolates were all identified by applying cultural characteristics and biochemical tests. The Polymerase Chain Reaction (PCR) was conducted to distinguish *algD, BLA-VIM, BLA-IMP*, and 16SrRNA genes. Moreover, the phenotypic method was used to detect hemolysin. The disk diffusion technique was applied to screen clinical isolates for eight antimicrobial agents. The PCR results showed all isolates to be positive for *algD* and negative for *BLA-VIM* and *BLA-IMP* genes. Hemolysin and multidrug resistance prevalence was 100% and 76%, respectively. Furthermore, Meropenem proved to be the most efficient antibiotic against clinical isolates. Alginate and hemolysin are considered significant virulence factors for *P. aeruginosa*, playing a key role in triggering diseases and tissue or skin lesions. The emergence of Multidrug Resistant (MDR) isolates indicates that developing antibiotic stewardship in our regional community hospital is a top priority. Infection control measures could help control the distribution of virulence genes in *P. aeruginosa* isolates. Moreover, regular observation is needed to decrease public health threats, distributing virulence factors and Imipenem-resistance patterns in clinical isolates of *P. aeruginosa*.

## INTRODUCTION

*P. aeruginosa* forms several secreted and cell-related virulence factors with a role in infection pathogenesis [1]. Forming intractable biofilm and secreting myriads of virulent factors such as Type III secretion (TTSS) effectors, LasB elastase, LasA protease, pyoverdin, pyocyanin, and alginate leads to the development of *P. aeruginosa* pathogenicity [2–5]. Mucoid strains may yield biofilms, representing communities of attached microorganisms on a surface. Biofilms have a crucial part in infectious diseases. Further, they have a favorable antibiotic resistance, with their matrix playing a major role [6–8].

Producing mucoid colonies using *P. aeruginosa* strains with alginate defends the organism against antimicrobials and the host's immune system response; thus, it facilitates the pulmonary system's chronic inflammation [3]. Alginates, like lipopolysaccharide (LPS), act in the adherence of the bacterium to the respiratory epithelium [9]. They also operate as a barrier to certain antibiotics. In other words, alginate formation reduces aminoglycosides' absorption and early bacterial effect. Moreover, alginate hinders the diffusion of positively charged hydrophilic medicines [7, 10]. The expression of the *algD* gene is influenced by environmental factors, including nitrogen/carbon/phosphate limitation, slow growth rate, and oxygen concentration [11, 12].

Alginate, structurally, is a simple linear polysaccharide; it has a high molecular weight, and it contains two uronic acids: β-D-mannuronic acid and its C5 epimer, α-Lguluronic acid. It is a capsule-like exopolysaccharide in *P. aeruginosa* and loosely adheres to *P. aeruginosa* cells, thus being found in the culture supernatant [13].

In *P. aeruginosa*, antibiotic resistance involves several processes, such as overexpressing active efflux systems, producing modifying enzymes, and reducing external membrane permeability [14].

*P. aeruginosa* infections grow intrinsically and could display attained resistance to various routinely prescribed antimicrobial medications. Furthermore, they are usually difficult to be treated owing to the advent of multidrug-resistant *P. aeruginosa* isolates [15]. Many studies have used the term multidrug-resistant *P. aeruginosa* to describe isolates resistant to at least three types of antimicrobial drugs, most commonly carbapenems, aminoglycosides, antipse1udomonal penicillins, cephalosporins, and quinolones [16].

The primary antimicrobial agents to treat *P. aeruginosa* infections are carbapenems, such as Imipenem, doripenem, and Meropenem [17]. Such antibiotics are grouped as β-lactam antibiotics. Carbapenems have a broad spectrum of activity as a major drug to treat *P. aeruginosa* infections. However, carbapenem hydrolyzing enzymes, including MBLs, can effectively hydrolyze all beta-lactam antibiotics, except for monobactams [18].

Mechanisms of carbapenem resistance occur for several reasons, including reduced outer membrane permeability, decreased expression or defect in outer membrane porines including OprD, presence of chromosomal AmpC beta-lactamase gene, production of beta-lactamase enzymes, and increase in the activity of pump efflux systems [19–21]. Therefore, the present descriptive research is performed to assess the profiles of antibiotic resistance and the prevalence of *BLA-VIM* and *BLA-IMP* genes in imipenem-resistant *P. aeruginosa*, as well as detecting hemolysin factor and alginate encoding genes from clinical isolates.

## MATERIAL AND METHODS

### P. aeruginosa Isolation and identification

Fifty *P. aeruginosa* isolates were obtained from sputum and wound samples of admitted patients in the Teaching Rizgari Hospital between January and April 2019. Isolates were identified based on the standard bacteriological approaches, such as colony morphology, pyocyanin pigment production, Gram staining, growth at 44℃, and biochemical tests [22]. Moreover, the identification was confirmed by 16S rRNA [23]. In addition, some bacteria isolates were stored at -70℃ in a microtube containing tryptic soy broth (TSB) and 20% glycerol for further studies [24].

### Antimicrobial susceptibility testing

Based on the Clinical and Laboratory Standards Institute (CLSI) guideline [25], antimicrobial susceptibility was defined on Mueller-Hinton agar via the Kirby Bauer disk diffusion assay. The susceptibility profiles were determined for eight antibiotics, namely Piperacillin/Tazobactam (100/10 µg), Piperacillin (PIP, 100 µg), Gentamicin (GEN, 10 µg), Ceftazidime (CAZ, 30 µg), Imipenem (IPM, 10 µg), Amikacin (AMK, 30 µg), Ciprofloxacin (CIP, 5 µg), and Meropenem (10 µg).

In each antimicrobial susceptibility assay, *P. aeruginosa* ATCC 27835 was applied as quality control. Based on the CLSI criteria and the manufacturer's protocols, the results were considered resistant or susceptible.

### Hemolysin detection

Bacterial isolated colonies were all plated (Sheep blood agar 5%) at 37℃ for 24h to detect hemolysins. The lysis of red blood cells (clear zone) was produced close to the cultured colonies following incubation, indicating positivity for hemolysins [26].

### Bacterial genomic DNA extraction

In Tryptic Soy Broth (Merck, Germany), bacterial strains were sub-cultured, then incubated at 37℃ for 48 h. Following the manufacturer's instructions, the DNA extraction kit (Geneaid/Korea) was used to extract the DNA from *P. aeruginosa* colonies. The extracted DNA was then subjected to PCR reactions, targeting *BLA-VIM, BLA-IMP*, and *algD* genes.

### Molecular analysis of BLA-VIM, BLA-IMP, and algD by polymerase chain reaction (PCR)

Table 1 shows how primer sequences and amplification protocols work [27, 28]. Then to identify *BLA-VIM* and *BLA-IMP*, Multiplex PCR was used as a method to amplify specific gene target sequences in a thermocycler (Techne/UK). The master mix preparation was executed in a total volume of 25 µl (12.5 µl Gotaq Green Master Mix (Promega/USA), 3 µl of genomic DNA, 1 µl for each *BLA-IMP* and *BLA-VIM* primer, and 5.5 µl nuclease-free water). For *BLA-VIM* and *BLA-IMP* amplification reactions, the mixtures were heated for 4 min at 95℃ before thermocycling, DNA denaturation for 30 sec at 94℃, primer annelation for 1 min at 60℃, and extensions for 1 min at 72℃. In the next step, the mixtures were kept at 72℃ for 6 min after finishing 30 cycles.

**Table 1:** The oligonucleotide sequences for the antibiotic-resistant and virulence genes.

Target gene	Primer sequences (5' – 3')	PCR product size	Reference
***BLA-IMPF***	5'GGCATAGAGTGGCTTAATTCTC3'	250 bp	Saberi *et al*., 2017 [27]
***BLA-IMPR***	5'GGCCAAGCTTCTATATTTGC3'		
***BLA-VIMF***	5'CAAATTGGACTTCCTGTAACG3'	273 bp	
***BLA-VIMR***	5'TATAGAGGTGGGCCATTCAG3'		
***algDF***	5'ATG CGA ATC AGC ATC TTT GGT3'	1310 bp	Benie *et al*., 2017 [28]
***algDR***	5'CTA CCA GCA GAT GCC CTC GGC3'		

For *algD* identification gene, all strains were checked by Uniplex PCR. The PCR premix for the *algD* gene was in a total volume of 25 µl, 12.5 µl (Gotaq Green Master Mix (Promega/USA), 3 µl genomic DNA, 1.5 µl for each primer, and 6.5 µl nuclease-free water). The PCR was performed as initial denaturation for 5 min at 94℃. Next, the 35-cycle amplification for 35 secs at 94℃ was executed followed by an annellation for 1 min at 61℃, an extension at 72℃ for 1 min, and, lastly, another extension for 10 min at 72℃ (Table 1).

The PCR product amplicons were finally analyzed using agarose gel electrophoresis (2%), and the DNA was stained with Safe dye (Bioland/USA). In addition, the presence and absence of the virulence and antibiotic-resistant genes were determined using a UV transilluminator (Syngene/UK). Also, the PCR product band for *BLA-VIM, BLA-IMP*, and *algD* genes was identified on the gel compared to the standard DNA ladder/1kb (Norgenbiotek/Canada).

## RESULTS

**Figure 1:**
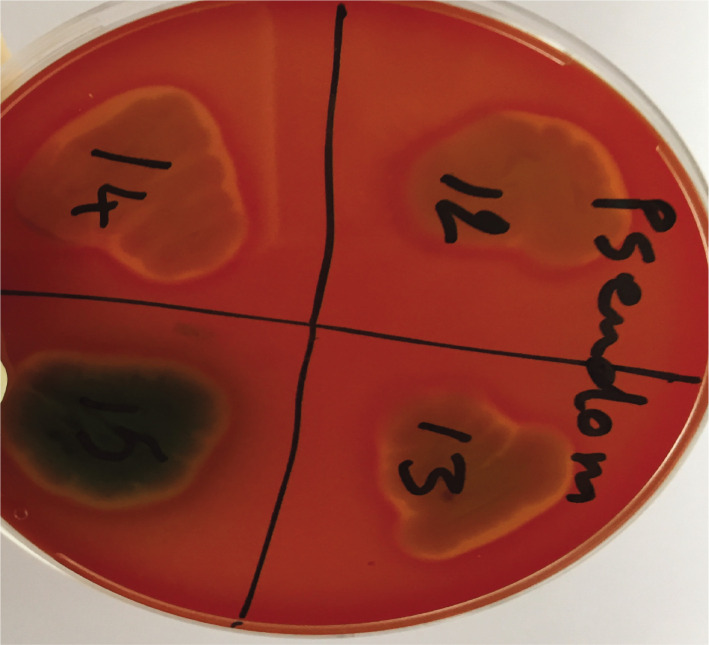
Clear beta-type hemolysis around the P. aeruginosa colonies on blood agar media.

In the present cross-sectional descriptive research, 45 bacterial isolates from wound infections and 5 strain isolates from sputum samples were collected. The clinical specimens were then inoculated into the routine and selective culture media for *P. aeruginosa* (Figure 1). Biochemical tests were performed to identify the colonies, which was confirmed by 16SrRNA [27].

**Figure 2:**
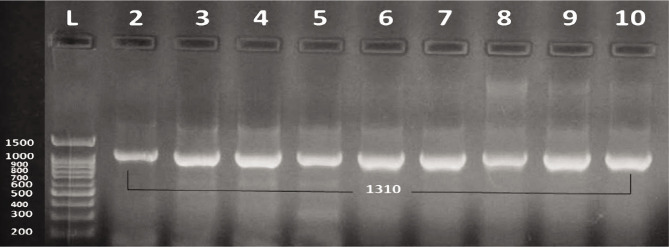
Electrophoresis of the amplicon products for the virulence gene in P. aeruginosa; Lane: 1 ladder of 1kb, Lane 2-10: algD (1310 bp) gene.

The PCR results revealed that all bacterial isolates carried the alg D gene, and none was positive for *BLA-VIM* and *BLA-IMP* genes (Figure 2).

All isolates appeared to be resistant to Piperacillin, Ceftazidime, and Piperacillin/Tazobactam in the current research. In contrast, the resistance to Imipenem, Meropenem, Amikacin, Gentamicin, Tobramycin, and Ciprofloxacin, respectively were 5%, 5%, 9%, 15%, 15%, and 9%. In contrast, intermediate resistance to Imipenem and Gentamicin was 16% and 3%. In addition, 76% of the isolates were multidrug-resistant [38].

## DISCUSSION

The frequency of the *algD* gene was 100%. This finding was in line with earlier studies, indicating that the gene exists in about all specimens [29].

The researchers [30–32] reported 98%, 95.24%, and 92.3% isolates to be positive for the *algD* gene, respectively.

Many hypotheses have attempted to clarify biofilm resistance (poor antibiotic penetration, nutrient limitation, high cell density, slow growth, adaptive stress response); however, no data exist explaining this phenomenon to date [33]. Fazlul has reported 48.33% and 7.5% of *P. aeruginosa* isolates to produce Hemolysin and Metallo beta-lactamase [26].

VIM and *BLA-IMP* genes were not detected; this finding was in line with that of ALjaafreha. Further, Shahi has reported none of the isolates to be *BLA-VIM* genes positive. According to previous and current studies, the differences observed in the incidence of *BLA-VIM* and *BLA-IMP* genes can be explained by the use of Imipenem and improper application of antibiotics in different regions [29, 34].

In *Pseudomonas aeruginosa*, mechanisms such as OprD, AmpC, and MexAB, as non-carbapenemase resistance mechanisms, may develop carbapenem resistance [35–38]. Pump efflux systems play an important role in the antibiotic resistance of *Pseudomonas aeruginosa* isolates to various antibiotics. *Pseudomonas aeruginosa* has the potential to express 12 types of multidrug efflux pumps called Mex. Mex efflux systems belong to the Resistance-Nodulation-Division (RND) family. MexAB-OprM is the only secretory pump found in all *Pseudomonas aeruginosa* isolates and causes the inherent resistance of these bacteria to antibiotics. Ertapenem, Meropenem and Doripenem are substrates of the MexAB-OprM efflux system. Therefore, increasing the expression of MexAB-OprM pump efflux system plays an important role in the antibiotic resistance of *Pseudomonas aeruginosa* isolates to beta-lactam antibiotics, especially carbapenems [39, 40]. Another resistance mechanism in *Pseudomonas aeruginosa* is AmpC cephalosporins, a chromosomally encoded enzyme whose basic expression is resistance to beta-lactams except for cefpime, ceftazidime and carbapenems. Therefore, meropenem and imipenem resistant profiles can be created due to increased expression of MexAB-OprM flow pump genes, decreased expression or purine expression of OprD, and overexpression of AmpC carbapenemase, respectively. In this regard, some authors have suggested the possibility of inferring the mechanism of resistance from the antibiogram [41–43].

With the spread of ESBL-producing gram-negative bacteria and carbapenemase-producing organisms that cause infection in patients in intensive care units, the use of carbapenems seems necessary [44].

All isolates in the current research were resistant to Tazobactam/Piperacillin. Metallo beta-lactamase *P. aeruginosa* were resistant to different antimicrobial agents, except for Tazobactam/Piperacillin. The multidrug-resistant phenotype is anticipated in Metallo beta-lactamase producers since they hydrolyze all beta-lactams, apart from aztreonam, and are related to gene cassettes, protecting various resistance genes [45].

To identify regulatory genes and structural changes in Carbapenem-resistant encoded proteins, further studies should be performed in addition to studies in the field of identification and expression of genes of this resistance. Extensive studies should also be performed to further identify the causes of the regular occurrence of virulence-related genes in MDR isolates.

## CONCLUSIONS

In this study, the increase and spread of potentially very pathogenic and Carbapenem-resistant strains incorporated with multidrug-resistant patterns are concerned since a probable result would have increased clinical severity and would be accompanied by significant restrictions in antibiotic therapy. Imipenem resistance isolates comprise virulence factors, including hemolysin and alginate, responsible for serious infections.
